# Upregulation of Sox2 Following Saracatinib Treatment Contributes to a Resistant Phenotype in Colorectal Cancer Cells under Growth Factor-Supplemented Conditions

**DOI:** 10.32604/or.2026.074140

**Published:** 2026-04-22

**Authors:** Chanwoong Yoon, Euihyeon Na, Min Joo Choi, Sang-Pil Yoon

**Affiliations:** 1 Interdisciplinary Postgraduate Program in Biomedical Engineering, Graduate School, Jeju National University, Jeju, Republic of Korea; 2 Department of Psychiatry, Soonchunhyang University Cheonan Hospital, Soonchunhyang University College of Medicine, Cheonan, Republic of Korea; 3 Department of Anatomy, College of Medicine, Jeju National University, Jeju, Republic of Korea

**Keywords:** Src inhibitor, drug resistance, epithelial-mesenchymal transition, sex determining region Y-box-2, colorectal cancer

## Abstract

**Objective:**

Increased Src kinase activity is known to correlate with cancer progression and poor prognosis, indicating that Src plays a central role in cell migration and invasion. In this study, we investigated the effects of saracatinib, a Src kinase inhibitor, under anoikis-resistant conditions in colorectal cancer cells.

**Methods:**

Wild-type and 5-fluorouracil-resistance acquired SNU-C5 colorectal cancer cells were cultured in both monolayer and spheroid systems under fetal bovine serum (FBS) or growth factor (GF) supplemented conditions. Cell viability assay, flow cytometry, wound healing assay, spheroid formation and morphometric analysis, and Western blotting were performed using both adherent cells and spheroids.

**Results:**

Saracatinib significantly reduced cell viability and migration in both cell lines, predominantly through the induction of apoptosis. Spheroid formation was less efficient under GF-supplemented conditions than under FBS-supplemented conditions. The anti-cancer effects of saracatinib were mediated through inhibition of p38 mitogen-activated protein kinase (MAPK), extracellular signal-regulated protein kinase (ERK), or epidermal growth factor receptor (EGFR) signaling pathways. Although most cancer stem cell (CSC) markers were suppressed by saracatinib, expression of sex determining region Y-box-2 (Sox2) was paradoxically increased in monolayer cultures. Upon re-treatment with saracatinib, Sox2-upregulated cells formed larger spheroids under GF-supplemented conditions compared with wild-type cells.

**Conclusions:**

Saracatinib exerts anti-cancer effects in colorectal cancer cells by downregulating MAPKs, EGFR, and CSC-associated markers. However, paradoxical upregulation of Sox2 influenced spheroid formation under GF-supplemented conditions, suggesting that Sox2 may contribute to drug resistance or recurrence in colorectal cancers.

## 1 Introduction

Colorectal cancer (CRC) remains one of the leading causes of cancer and cancer-related mortality worldwide. The effectiveness of chemotherapy is frequently compromised by the development of multi-drug resistance to 5-fluorouracil (5-FU)-based chemo-therapeutic agents [1,2]. Despite research efforts, the molecular mechanisms underlying drug resistance in CRC have not yet been fully elucidated. Dysregulation of protein kinase B (Akt) and extracellular signal-regulated protein kinase (ERK) has been implicated [3]. SNU-C5 cells that have acquired resistance to 5-FU (SNU-C5/5-FUR) cells exhibited enhanced drug efflux mediated by ATP-binding cassette subfamily G member 2 (ABCG2) along with reduced activation of ERK compared with parental SNU-C5 cells [4]. In addition, ERK activation is crucial for the anti-cancer activity of 5-FU in SNU-C5 CRC cells [5].

Cancer stem cell (CSC) can be identified by the expression of specific markers such as cluster of differentiation (CD) 44 and transcription factors like octamer binding transcription factor-4 (Oct4), sex determining region Y-box-2 (Sox2), cMyc, Krüppel-like factor 4 (Klf4), and Nanog [6,7]. The epithelial-mesenchymal transition (EMT) is thought to promote the acquisition of CSC-like properties in non-CSCs [8]. Because EMT-induced cells can form spheroids under anchorage-independent conditions [9,10], spheroid formation assays are widely used to assess CSC-like characteristics and to recapitulate the 3-dimensional (3D) *in vivo* microenvironment. Furthermore, growth factor (GF)-free fetal bovine serum (FBS)-supplemented conditions are considered a more efficient and cost-effective approach for *in vitro* CSC enrichment than GF-supplemented conditions [11,12].

Src, a protein-tyrosine kinase [13], is closely associated with mitogen-activated protein kinase (MAPK) signaling, or epidermal growth factor (EGF) receptor (EGFR) activity [14,15]. Interactions between Src and MAPKs [16] or among Src, EGFR, and Akt [17] promote CRC migration, invasion, and proliferation. These findings suggest that the anti-cancer effects of saracatinib, a Src kinase inhibitor, may be mediated through inhibition of p38 [15] or ERK [16] signaling pathways in CRCs. Nevertheless, the precise mechanisms of Src kinase inhibitors in CRCs remain incompletely understood, particularly under microenvironmental conditions that more closely resemble *in vivo* tumor growth. CD44^+^ CSCs derived from spheroids exhibit Sox2 expression with reduced p38 and ERK activity [18]. Moreover, 5-FU treatment decreases the CD44^+^ CSC population and reduces Sox2 expression and p38 phosphorylation [19].

Because saracatinib has been reported not to affect the efficacy of 5-FU [20], wild-type and 5-FU-resistance acquired SNU-C5 CRC cell lines represent suitable models for exploring the mechanisms underlying the anti-cancer effects of saracatinib. In this study, we evaluated the anti-cancer activity of saracatinib in CRCs under different supplementation conditions and assessed whether spheroid formation is influenced by external supplements. We also investigated potential mechanistic interactions among Src, MAPKs, EGFR, and CSC-associated markers.

## 2 Materials and Methods

### 2.1 Cell Culture (2D Culture)

SNU-C5 (the 50% cytotoxic concentration (CC_50_) against 5-FU = 5 μM) and SNU-C5/5-FUR (CC_50_ against 5-FU = 140 μM) cell lines were provided by the Korean Cell Line Bank (#0000C5, Seoul, Republic of Korea) and the Research Center for Resistant Cells (Chosun University, Gwangju, Republic of Korea), respectively. Parental SNU-C5 cells were authenticated by short tandem repeat profiling and were confirmed mycoplasma-free by the supplier, and both cell lines were routinely testing negative for mycoplasma contamination using Myco-Visible mycoplasma rapid test kit (#3050901; MP Biomedicals, Solon, OH, USA). After thawing, the cells were cultured in RPMI-1640 medium (#10-040-CV) supplemented with 10% FBS (#35-015-CV) and 1% penicillin-streptomycin (#30-002-CI) at 37°C in a 5% CO_2_ incubator [4,5]. Cells from up to 10 subcultures were used in the following experiments. All cell culture reagents used were purchased from Corning Inc. (Corning, NY, USA).

### 2.2 Cell Viability Assay

The impact of saracatinib on cell viability was assessed using an MTT reduction assay, as previously described [4,5]. SNU-C5 (2 × 10^3^ cells/well) and SNU-C5/5-FUR (5 × 10^3^ cells/well) cells were seeded in 96-well plates. Cells were incubated with saracatinib (#11497, Cayman Chemical, Ann Arbor, MI, USA; 0, 0.1, 1, 10, and 100 μM) for 3 days, after which 10 μL of MTT solution (#M6494, Thermo Fisher Scientific, Waltham, MA, USA; 5 mg/mL in phosphate-buffered saline), and incubated for 3-h. The resulting formazan crystals were dissolved in dimethyl sulfoxide (DMSO) for 15 min, and absorbance was measured spectrophotometrically at 595 nm using a VERSAmax microplate reader (Molecular Devices Korea LLC.; Seoul, Republic of Korea) with 620 nm as a background reference. Absorbance values for vehicle-treated controls were considered indicate of 100% viability.

### 2.3 Flow Cytometry

SNU-C5 and SNU-C5/5-FUR cells were incubated with or without saracatinib (10 μM and 15 μM, respectively) for 3 days, and then subjected to flow cytometry, as previously described [8]. Cell death was determined by staining suspended cells with 5 μL annexin V-FITC and 5 μL propidium iodide for 15 min at room temperature in the dark using the ExWay Annexin V-FITC Apoptosis Detection Kit (#K29100, KOMA Biotech; Seoul, Republic of Korea). Samples were processed using FACSCalibur^TM^ system, and data were analyzed using FACStation software version 6.0 (BD Biosicences, San Jose, CA, USA).

### 2.4 Wound Healing Assay

Cell migration was assessed using the scratch assay. SNU-C5 (2 × 10^5^ cells) and SNU-C5/5-FUR (5 × 10^5^ cells) cells were seeded in six-well plates and, after reaching 90% confluence, were subjected serum-starvation overnight in RPMI 1640 medium (#10-040-CV). Scratches were made with a 20-μL pipette tip, and cells were then incubated with or without saracatinib (10 μM for SNU-C5 and 15 μM for SNU-C5/5-FUR cells). Wound closure was monitored every 24 h for up to 3 days. Images of the scratches were captured using a DP70 digital camera attached to a BX-50 light microscope (Olympus, Tokyo, Japan). Wound areas were quantified using Image-J software (version 1.38 with the MRI wound healing tool, http://rsb.info.nih.gov/ij/). Initial wound areas were considered 100%. Experiments were conducted in at least triplicate, and wound areas were measured at three different positions.

### 2.5 Spheroid Culture (3D Culture)

Ultra-low attachment 96-well plates were used to create anchorage-independent growth conditions as previously described [12]. SNU-C5 and SNU-C5/5-FUR cells (respective cell densities are given in the figures) were initially seeded in round-bottom plates (#7007, Corning), and treated with saracatinib (0, 0.1, 1, 10, and 100 µM) in DMEM/F12 medium (#10-090-CV, Corning) containing 1% B27 supplement (#17504-044, Thermo Fisher Scientific), 20 ng/mL EGF (#PHG0311, Gibco, Thermo Fisher Scientific) and 20 ng/mL bFGF (#13256029, Gibco, Thermo Fisher Scientific) for 15 days (the GF group), or in the same medium supplemented with FBS (the FBS group). The medium was changed every 5 days. Spheroidogenesis was assessed in flat-bottom plates (#3474, Corning) with or without saracatinib (30 µM) for up to 21 days with medium replacement every 7 days. SNU-C5 and SNU-C5/5-FUR cells were seeded in conditions supplemented with FBS and GF, respectively.

Spheroid diameters were analyzed 15 days after seeding in round-bottom plates or in 4 fields per well 21 days after seeding in flat-bottom plates. Spheroid images were captured using a DP70 digital camera attached to a BX-50 light microscope (Olympus). Quantification was performed using iSolution Lite software (for window 10; IMT Inc., Irvine, CA, USA).

Wild-type and Sox2-upregulated SNU-C5 and SNU-C5/5-FUR cells were seeded (the respective cell densities are shown in the figures) in round-bottom plates. After addition of saracatinib (0, 0.1, 1, 10, and 100 µM), cells were cultured for 15 days in medium supplemented with FBS and GF. Spheroid growth patterns were evaluated by measuring their diameters after culture with or without saracatinib (30 µM) for 15 days with medium changes every 5 days.

### 2.6 Western Blot Analysis

SNU-C5 and SNU-C5/5-FUR cells were cultured for 3 days as monolayers (2D) or for 15 days as spheroids (3D) in medium supplemented with FBS. Saracatinib was administered to SNU-C5 and SNU-C5/5-FUR cells in 2D cultures at a concentration of 10 and 15 μM, respectively, and to both cell lines in 3D cultures at a concentration of 30 μM.

Proteins in cell lysates were quantified and subjected to electrophoresis, as previously described [4,5]. For intracellular proteins extraction, cells and spheroids were treated using M-PER mammalian protein extraction reagent (#78501, Thermo Fisher Scientific) containing 1% protease inhibitor cocktail (#ab201111, Abcam), 0.5% phosphatase inhibitor cocktail I (#ab201112, Abcam), and II (#ab201113, Abcam). Protein concentrations were measured using a BCA protein assay kit (#23225, Thermo Fisher Scientific). Protein (30 μg/mL) electrophoresis was performed with a TGX Stain-Free FastCast^TM^ Acrylamide Starter Kit (#1610181 and #1610185) Bio-Rad Laboratories Inc., Seoul, Republic of Korea), using a Tris/glycine buffer system. Proteins were transferred to polyvinylidene fluoride membranes (#162-0176; Bio-Rad Laboratories, Hercules, CA, USA), which were blocked at room temperature with 5% skim milk (#SKI500, LPS solution, Daejeon, Republic of Korea) for 1 h, incubated overnight with primary antibodies (Table 1) at 4°C, washed, and treated with peroxidase-conjugated anti-mouse or anti-rabbit IgG antibodies (#PI-2000 and #PI-1000; Vector Laboratories Inc., Burlingame, CA, USA) for 1 h at room temperature. Proteins were then detected using Western Lightning Chemiluminescence Reagent (#104001EA, PerkinElmer Inc.; Waltham, MA, USA). Anti-GAPDH antibody was used as a loading control after membrane stripping. Bands were visualized using the Azure^TM^ c300 chemiluminescence imaging system and quantified using AzureSpot analysis software (version 14.2; Azure Biosystems Inc., Dublin, CA, USA).

**Table 1:** Primary antibodies used in the experiment.

	Name (Catalog Number)	Company	Dilution
Primary antibodies	Akt (#sc-8312)	Santa Cruz Biotechnology (Santa Cruz, CA, USA)	1:1000
	cMyc (9E10; #sc-40)		1:1000
	ERK (#sc-93)		1:2000
	GAPDH (#sc-47724)		1:2000
	Oct3/4 (#sc-5279)		1:1000
	Nanog (#sc-293121)		1:1000
	Phosphorylated Akt (#sc-7985)		1:1000
	Pan-Ras (#sc-166691)		1:1000
	Sox2 (#sc-365823)		1:1000
	CD44 (#ab157107)	Abcam (Cambridge, MA, USA)	1:1000
	EGFR (#ab52894)		1:1000
	Klf4 (#4038)		1:1000
	Phospho-ERK (#4370)	Cell Signaling Tech (Beverley, MA, USA)	1:1000
	p38 MAPK (#9212)		1:1000
	Phospho-p38 MAPK (#9211)		1:1000

### 2.7 Statistical Analysis

Statistical significance was determined using paired Student’s *t*-test or one-way analysis of variance (ANOVA) with post-hoc test in MS Excel 2016. Results were derived from at least three independent experiments and are presented as mean values ± standard deviation (SD). *p* value < 0.05 was considered statistically significant.

## 3 Results

### 3.1 Anti-Cancer Effects of Saracatinib on CRC Cells

**Figure 1:**
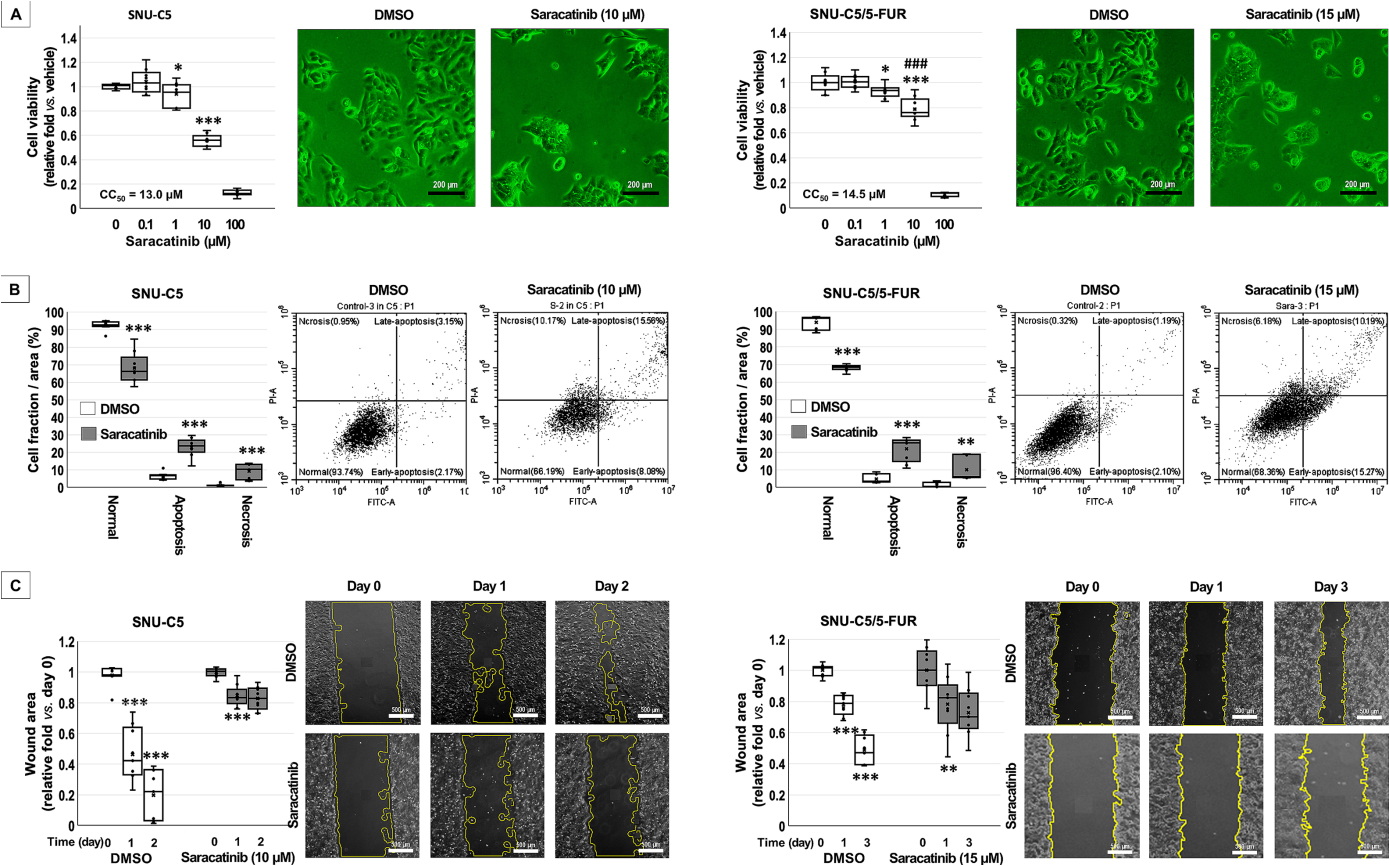
Anti-cancer effects of saracatinib in colorectal cancer cells. (**A**) Cells were treated with vehicle (dimethyl sulfoxide, DMSO) or saracatinib (indicated concentrations in figures) for 3 days. Representative images are shown. Cell viability was assessed using the MTT assay. Data (fold changes) are shown as mean ± SD. *p < 0.05, ***p < 0.001 vs. DMSO; ^###^*p* < 0.001 vs. SNU-C5 cells. (**B**) Cell death was evaluated using annexin V/propidium iodide staining followed by flow cytometry after treatment with DMSO or saracatinib (10 μM for SNU-C5 and 15 μM for SNU-C5/5-FUR) for 3 days. The percentages of normal, apoptotic, and necrotic cells are presented as mean ± SD. **p < 0.01 and ***p < 0.001 vs. DMSO. (**C**) Changes in wound area following treatment with DMSO or saracatinib (as described in (**B**)) were measured over time, as indicated, with representative images. Data (fold changes) are presented as mean ± SD. **p < 0.01, ***p < 0.001 vs. the previous time point.

The MTT assay (Fig. 1A) revealed significant changes at 1 μM in SNU-C5 (93.6 ± 9.7%, *p* = 0.0479) and SNU-C5/5-FUR (93.1 ± 5.2%, *p* = 0.0250) cells. At 10 μM, a significant difference between the cell lines (*p* < 0.001) was observed, with viability values of 55.6 ± 5.0% for SNU-C5 and 78.8 ± 9.1% for SNU-C5/5-FUR cells. Saracatinib concentrations close to CC_50_ were therefore used for subsequent experiments: 10 μM for SNU-C5 and 15 μM for SNU-C5/5-FUR cells.

Flow cytometry (Fig. 1B) demonstrated that saracatinib treatment significantly increased cell death compared to vehicle-treated controls. In SNU-C5 and SNU-C5/5-FUR cells, the proportion of apoptotic cells increased from 6.33 ± 2.02% to 22.79 ± 5.24% and from 4.73 ± 2.54% to 21.93 ± 6.71%, respectively (*p* < 0.001 for both). The proportion of necrotic cells increased from 1.18 ± 0.66% to 9.14 ± 4.10% in SNU-C5 cells (*p* = 0.0002) and from 1.25 ± 1.37% to 10.02 ± 6.55% in SNU-C5/5-FUR cells (*p* = 0.0017).

The scratch assay revealed a marked reduction in wound areas after saracatinib treatment (Fig. 1C). In vehicle-treated SNU-C5 and SNU-C5/5-FUR cells, migration increased significantly over time (*p* < 0.001 for both). In SNU-C5 cells, vehicle treatment reduced the wound area to 0.46 ± 0.06-fold and 0.20 ± 0.05-fold of baseline on days 1 and 2, respectively (*p* < 0.001). In contrast, saracatinib treatment maintained wound areas at 0.84 ± 0.02-fold (*p* < 0.001) and 0.83 ± 0.01-fold (*p* = 0.3108), respectively. In SNU-C5/5-FUR cells, vehicle treatment reduced wound areas to 0.78 ± 0.02-fold (*p* < 0.001) and 0.49 ± 0.03-fold (*p* < 0.001) on days 1 and 3, while saracatinib treatment resulted in wound areas of 0.78 ± 0.06-fold (*p* = 0.0057) and 0.73 ± 0.05-fold (*p* = 0.2512), respectively.

### 3.2 Spheroid Formation after Saracatinib Treatment in CRC Cells

**Figure 2:**
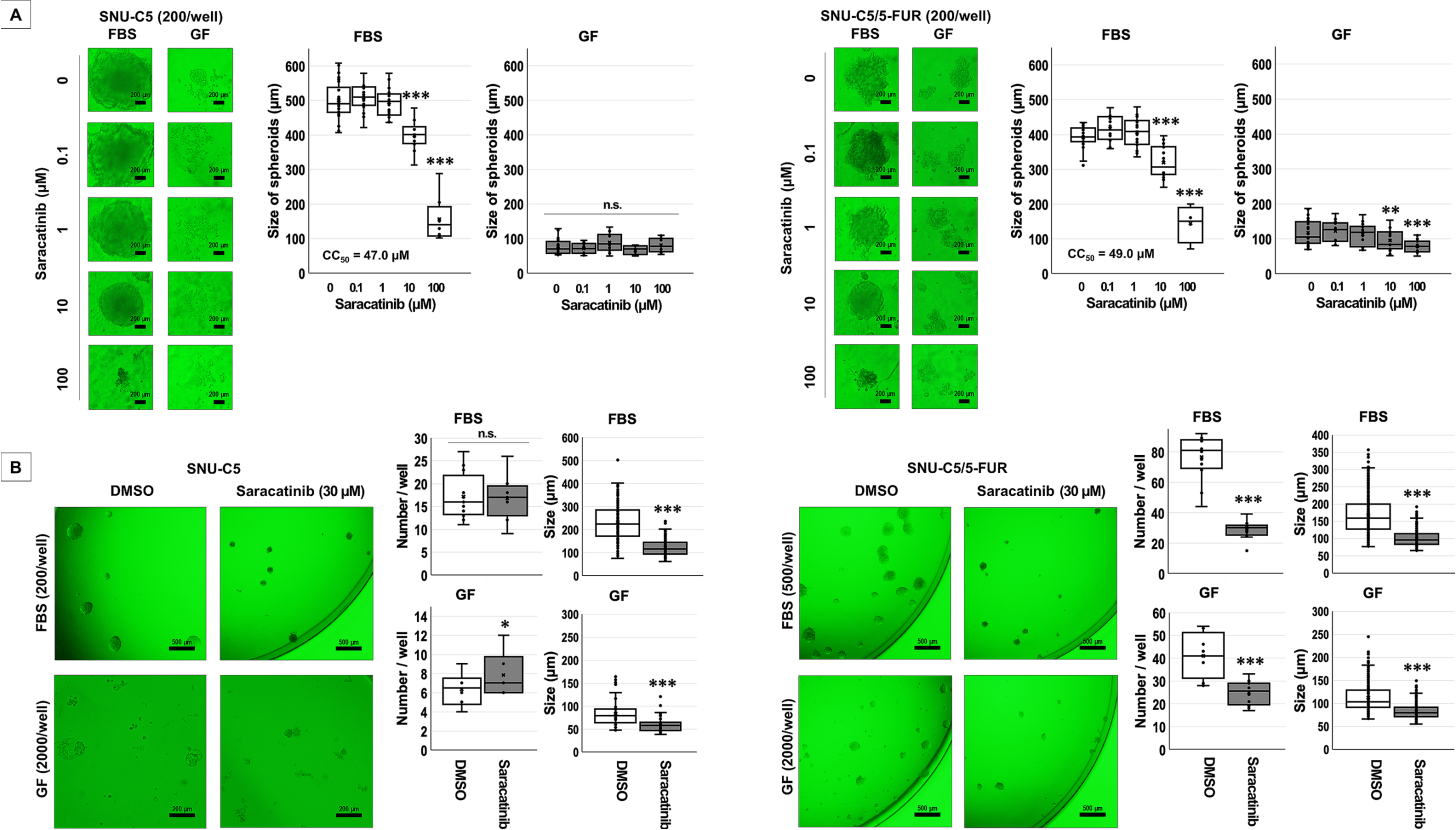
Anti-spheroidogenic effects of saracatinib in colorectal cancer cells under different supplementation conditions. (**A**) Spheroid formation was induced in ultra-low attachment round-bottom wells under fetal bovine serum (FBS)- or growth factor (GF)-supplemented conditions. Cells were treated with vehicle (dimethyl sulfoxide, DMSO) or saracatinib (indicated concentrations in figures) for 15 days. Representative images are shown. Spheroid sizes were measured and are expressed as mean ± SD. **p < 0.01, ***p < 0.001 vs. DMSO; n.s., not significant. (**B**) Spheroid formation was performed in ultra-low attachment flat-bottom wells under FBS- or GF-supplemented conditions. Cells were treated with DMSO or saracatinib (30 μM) for 21 days. Representative images are shown. Spheroid numbers and sizes were quantified and are presented as mean ± SD. *p < 0.05, ***p < 0.001 vs. DMSO; n.s., not significant.

The effects of saracatinib on spheroid formation were examined using a spheroid formation assay, with morphometric data presented in Table 2 and Fig. 2A. Under FBS-supplemented conditions, spheroid size in SNU-C5 decreased significantly with increasing saracatinib concentrations (*p* < 0.001). In contrast, no significant effect was observed under GF-supplemented conditions. In SNU-C5/5-FUR spheroids, saracatinib induced significant, dose-dependent reductions in spheroid size under both FBS- and GF-supplemented conditions (*p* < 0.001 for both). Based on these results, 30 μM saracatinib was selected for subsequent spheroid experiments in both cell lines.

**Table 2:** Spheroidogenesis following saracatinib treatment in SNU-C5 and SNU-C5/5-FUR cells supplemented with fetal bovine serum (FBS) or growth factor (GF)s.

A (Round Bottom)			SNU-C5		SNU-C5/5-FUR
		FBS (μm)	GF (μm)	FBS (μm)	GF (μm)
Saracatinib (μM)	0	500.4 ± 51.6	76.2 ± 22.4	391.8 ± 32.8 (^b^*p* < 0.001)	117.4 ± 36.2 (^b^*p* < 0.001)
	0.1	507.0 ± 39.2	70.9 ± 15.7	416.4 ± 38.0	121.7 ± 29.2
	1	493.8 ± 41.8	89.0 ± 26.3	406.2 ± 41.8	112.3 ± 31.4
	10	398.7 ± 39.0 (^a^*p* < 0.001)	66.1 ± 11.9	318.3 ± 44.4 (^a^*p* < 0.001)	94.5 ± 28.3 (^a^*p* = 0.0052)
	100	155.5 ± 63.3	80.6 ± 20.0 (^a^*p* = 0.2840)	143.3 ± 54.0	78.4 ± 17.7
**B (Flat Bottom)**			**SNU-C5**		**SNU-C5/5-FUR**
		**FBS (μm)**	**GF (μm)**	**FBS (μm)**	**GF (μm)**
Number	DMSO	17.2 ± 5.0	15.8 ± 4.7	76.4 ± 15.1	53.4 ± 6.7
	Saracatinib (30 μM)	16.8 ± 4.4 (^a^*p* = 0.4156)	18.6 ± 2.0 (^a^*p* = 0.0408)	28.6 ± 5.9 (^a^*p* < 0.001)	39.3 ± 6.5 (^a^*p* < 0.001)
Size	DMSO	235.2 ± 18.2	98.0 ± 8.6	166.9 ±12.3	126.9 ± 6.1
	Saracatinib (30 μM)	121.9 ± 13.0 (^a^*p* < 0.001)	75.8 ± 5.7 (^a^*p* < 0.001)	101.5 ± 3.9 (^a^*p* < 0.001)	94.2 ± 5.6 (^a^*p* < 0.001)

Note: Results are expressed as mean ± SD. ^a^p, vs. DMSO; ^b^p, vs. SNU-C5.

Because spheroid formation was inefficient under GF supplementation, the seeding density for both CRC cells was increased in flat-bottom ultra-low attachment wells (Table 2 and Fig. 2B). Although saracatinib increased the number of SNU-C5 spheroids under GF-supplemented conditions (*p* = 0.0408), it significantly reduced their mean size (*p* < 0.001). In contrast, saracatinib reduced both the number and size of SNU-C5/5-FUR spheroids under both FBS- and GF-supplemented conditions (*p* < 0.001 for all comparisons).

### 3.3 Changes in Interacting Proteins Following Saracatinib Exposure in 2D and 3D Cultures of CRC Cells

**Figure 3:**
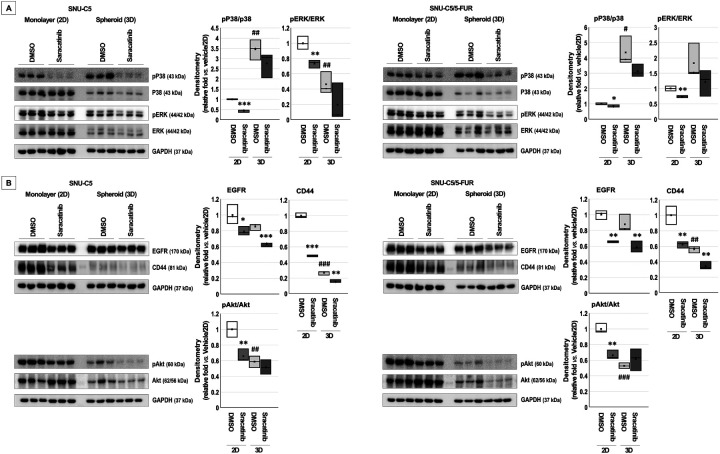
Proteins that interacted with Src following saracatinib treatment in colorectal cancer cells. (**A**) Cells were treated with dimethyl sulfoxide (DMSO) or saracatinib for 3 days in two-dimensional (2D) monolayer culture (10 μM for SNU-C5 and 15 μM for SNU-C5/5-FUR) or for 15 days in 3D spheroid culture (30 μM for both cell lines). Total protein extracts were subjected to immunoblotting for phosphor-p38 (pP38), p38, phosphor-ERK (pERK), and ERK. GAPDH served as the loading control. Band intensities were quantified using AzureSpot software, and activation ratios (phosphor/total protein) are represented as mean ± SD. *p < 0.05, **p < 0.01, ***p < 0.001 vs. DMSO; ^#^*p* < 0.05, ^##^*p* < 0.01 vs. DMSO/2D. (**B**) Immunoblotting was performed for EGFR, CD44, phosphor-Akt (pAkt), and Akt. GAPDH was used as the loading control. *p < 0.05, **p < 0.01, ***p < 0.001 vs. DMSO; ^##^*p* < 0.01, ^###^*p* < 0.001 vs. DMSO/2D.

Western blot analysis was performed to identify proteins interacting with Src (Table 3 and Fig. 3A). Saracatinib treatment reduced the activation (phorphor/total) of p38 and ERK under 2D and 3D culture conditions in both cell types. Basal p38 activation was higher in spheroids derived from both cell lines, and ERK activation was elevated in SNU-C5 spheroids.

**Table 3:** Densitometric analysis of Western blot results following saracatinib treatment in SNU-C5 and SNU-C5/5-FUR cells.

	Variables	SNU-C5				SNU-C5/5-FUR			
		Monolayer		Spheroid		Monolayer		Spheroid	
		DMSO	Saracatinib	DMSO	Saracatinib	DMSO	Saracatinib	DMSO	Saracatinib
A	p38	1.00 ± 0.02	0.43 ± 0.06 (^a^*p* < 0.001)	3.44 ± 0.49 (^b^*p* = 0.0067)	2.76 ± 0.60 (^a^*p* = 0.1019)	1.00 ± 0.06	0.84 ± 0.07 (^a^*p* = 0.0237)	4.35 ± 0.93 (^b^*p* = 0.0125)	3.13 ± 0.40 (^a^*p* = 0.0537)
	ERK	1.00 ± 0.09	0.73 ± 0.05 (^a^*p* = 0.0047)	1.62 ± 0.14 (^b^*p* = 0.0070)	1.60 ± 0.25 (^a^*p* = 0.4591)	1.00 ± 0.08	0.74 ± 0.04 (^a^*p* = 0.0031)	1.83 ± 0.56 (^b^*p* = 0.0628)	1.21 ± 0.42 (^a^*p* = 0.0989)
	EGFR	1.00 ± 0.12	0.79 ± 0.05 (^a^*p* = 0.0274)	0.85 ± 0.04 (^b^*p* = 0.0574)	0.63 ± 0.03 (^a^*p* = 0.0007)	1.00 ± 0.06	0.66 ± 0.01 (^a^*p* = 0.0047)	0.88 ± 0.11 (^b^*p* = 0.0798)	0.59 ± 0.07 (^a^*p* = 0.0091)
	CD44	1.00 ± 0.03	0.48 ± 0.01 (^a^*p* < 0.001)	0.27 ± 0.02 (^b^*p* < 0.001)	0.16 ± 0.02 (^a^*p* = 0.0018)	1.00 ± 0.12	0.62 ± 0.04 (^a^*p* = 0.0032)	0.56 ± 0.04 (^b^*p* = 0.0018)	0.34 ± 0.05 (^a^*p* = 0.0022)
	Akt	1.00 ± 0.10	0.66 ± 0.08 (^a^*p* = 0.0049)	0.59 ± 0.07 (^b^*p* = 0.0021)	0.52 ± 0.09 (^a^*p* = 0.1806)	1.00 ± 0.06	0.66 ± 0.06 (^a^*p* = 0.0011)	0.53 ± 0.04 (^b^*p* = 0.0002)	0.61 ± 0.14 (^a^*p* = 0.1822)
B	Oct3/4	1.00 ± 0.15	0.83 ± 0.09 (^a^*p* = 0.0895)	0.51 ± 0.11 (^b^*p* = 0.0056)	0.44 ± 0.03 (^a^*p* = 0.1927)	1.00 ± 0.09	0.69 ± 0.12 (^a^*p* = 0.0110)	0.51 ± 0.03 (^b^*p* = 0.0003)	0.26 ± 0.04 (^a^*p* = 0.0005)
	Sox2	1.00 ± 0.03	1.44 ± 0.12 (^a^*p* = 0.0017)	1.08 ± 0.14 (^b^*p* = 0.1934)	0.99 ± 0.06 (^a^*p* = 0.1825)	1.00 ± 0.12	1.19 ± 0.04 (^a^*p* = 0.0312)	1.19 ± 0.14 (^b^*p* = 0.0758)	1.03 ± 0.02 (^a^*p* = 0.0988)
	cMyc	1.00 ± 0.10	0.65 ± 0.10 (^a^*p* = 0.0064)	0.84 ± 0.29 (^b^*p* = 0.2133)	0.72 ± 0.06 (^a^*p* = 0.2706)	1.00 ± 0.11	0.59 ± 0.01 (^a^*p* = 0.0118)	0.40 ± 0.06 (^b^*p* = 0.0006)	0.34 ± 0.07 (^a^*p* = 0.1496)
	Klf4	1.00 ± 0.10	0.75 ± 0.13 (^a^*p* = 0.0295)	2.24 ± 0.54 (^b^*p* = 0.0302)	2.84 ± 0.62 (^a^*p* = 0.1352)	1.00 ± 0.03	0.56 ± 0.12 (^a^*p* = 0.0015)	1.26 ± 0.13 (^b^*p* = 0.0146)	1.47 ± 0.22 (^a^*p* = 0.1097)
	Nanog	1.00 ± 0.16	0.90 ± 0.12 (^a^*p* = 0.2090)	0.80 ± 0.14 (^b^*p* = 0.0934)	0.43 ± 0.08 (^a^*p* = 0.0089)	1.00 ± 0.07	0.85 ± 0.11 (^a^*p* = 0.0607)	1.25 ± 0.12 (^b^*p* = 0.0176)	1.10 ± 0.22 (^a^*p* = 0.1768)
	pan-Ras	1.00 ± 0.10	0.88 ± 0.02 (^a^*p* = 0.0858)	1.35 ± 0.11 (^b^*p* = 0.0071)	1.21 ± 0.14 (^a^*p* = 0.1195)	1.00 ± 0.05	0.83 ± 0.02 (^a^*p* = 0.0018)	1.32 ± 0.12 (^b^*p* = 0.0067)	1.15 ± 0.03 (^a^*p* = 0.0424)

Note: Results (fold changes) are expressed as mean ± SD. ^a^p, DMSO vs. Saracatinib; ^b^p, monolayer vs. spheroid.

Expression levels of EGFR and CD44 were markedly reduced after saracatinib treatment in both 2D and 3D cultures of CRC cells (Table 3 and Fig. 3B). Akt activation was substantially reduced by saracatinib in 2D cultures but remained unchanged in 3D cultures in both cell lines.

**Figure 4:**
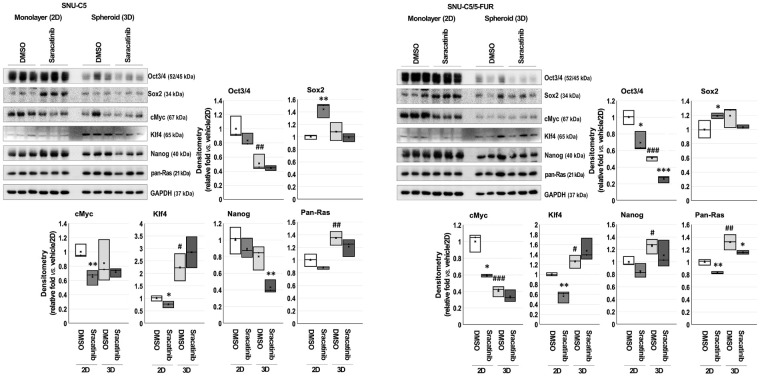
Effects of saracatinib on cancer stem cell (CSC) markers in colorectal cancer cells. Cells were treated with dimethyl sulfoxide (DMSO) or saracatinib for 3 days in a two-dimensional (2D) monolayer culture (10 μM for SNU-C5 and 15 μM for SNU-C5/5-FUR), and for 15 days in a 3D spheroid culture (30 μM for both). Total proteins extracts were immunoblotted for Oct3/4, Sox2, cMyc, Klf4, Nanog, and pan-Ras. GAPDH served as the loading control. Band intensities were quantified using AzureSpot software, and data are presented as mean ± SD. *p < 0.05, **p < 0.01, ***p < 0.001 vs. DMSO; ^#^*p* < 0.05, ^##^*p* < 0.01, ^###^*p* < 0.001 vs. DMSO/2D.

Saracatinib treatment reduced the expression of most CSC markers; however, Sox2 expression increased in 2D-cultured both CRC cells (Table 3 and Fig. 4). Compared with 2D cultures, basal expression levels of Klf4 and pan-Ras were significantly higher in spheroids from both cell lines, and Nanog increased in SNU-C5/5-FUR spheroids.

### 3.4 Spheroid Formation Following Saracatinib Treatment in Wild-Type and Sox2-Upregulated CRC Cells

**Figure 5:**
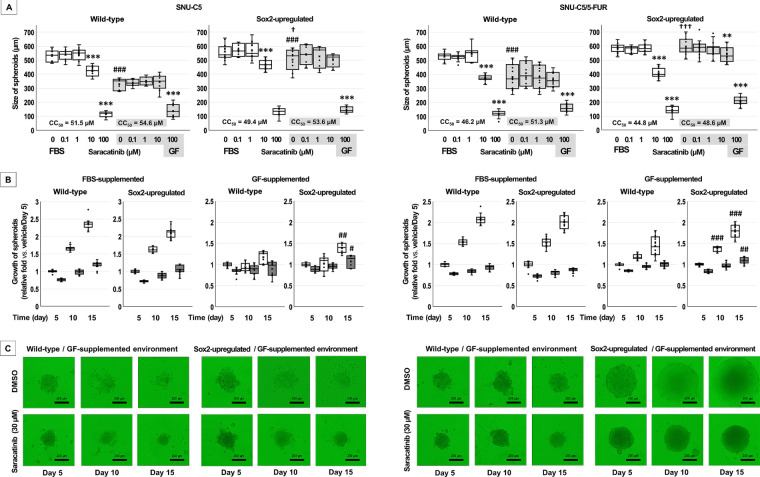
Spheroid formation by wild-type and Sox2-upregulated colorectal cancer cells under different supplementation conditions. (**A**) Spheroid formation was induced in ultra-low attachment round-bottom wells in DMEM/F12 medium supplemented with fetal bovine serum (FBS) or growth factor (GF). Wild-type and Sox2-upregualted cells were treated with dimethyl sulfoxide (DMSO) or the indicated concentrations of saracatinib for 15 days. Spheroid size was quantified and is presented as mean ± SD. **p < 0.01, ***p < 0.001 vs. DMSO; ^+^*p* < 0.05, ^+++^*p* < 0.001 vs. FBS; ^###^*p* < 0.001 vs. wild-type. (**B**) Wild-type and Sox2-upregulated spheroids were maintained for 15 days in ultra-low attachment round-bottom wells under FBS- or GF-supplemented conditions. Cells were exposed to DMSO or saracatinib (30 μM) for 15 days. Spheroid sizes were measured at 5-day intervals and are presented as mean ± SD. ^#^*p* < 0.05, ^##^*p* < 0.01, ^###^*p* < 0.001 vs. wild-type. (**C**) Representative spheroid images under GF-supplemented conditions.

Because Sox2 was the only CSC marker upregulated by saracatinib, Sox2-upregulated cells were used to compare spheroid formation with wild-type CRC cells (Table 4 and Fig. 5A). In this experimental setting, saracatinib did not significantly affect spheroid formation in either cell line. Notably, under GF-supplemented conditions, spheroids derived from Sox2-upregulated cells were larger than those derived from wild-type cells (*p* < 0.001 for both).

**Table 4:** Spheroidogenesis following saracatinib re-treatment in wild-type and Sox2-upregulated SNU-C5 and SNU-C5/5-FUR cells supplemented with fetal bovine serum (FBS) or growth factor (GF)s.

**A (Size)**		**Wild Type SNU-C5**		**Sox2-Upregulated SNU-C5**	
		**FBS** (μm)	**GF (μm)**	**FBS (μm)**	**GF (μm)**
Saracatinib (μM)	0	530.1 ± 42.5	328.3 ± 40.1 (^b^*p* < 0.001)	560.3 ± 54.8	502.3 ± 72.0 (^b^*p* = 0.0187)
	0.1	537.6 ± 36.5	339.3 ± 25.6	571.7 ± 42.5	542.5 ± 69.3
	1	540.7 ± 44.0	349.7 ± 28.7	567.6 ± 58.6	525.6 ± 76.8
	10	422.2 ± 36.4 (^a^*p* < 0.001)	343.4 ± 56.2	465.8 ± 36.1 (^a^*p* < 0.001)	499.9 ± 45.5
	100	114.1 ± 22.2	136.1 ± 47.6 (^a^*p* < 0.001)	131.8 ± 32.1	146.6 ± 22.5 (^a^*p* < 0.001)
		**Wild-Type SNU-C5/5-FUR**		**Sox2-Upregulated SNU-C5/5-FUR**	
		**FBS (μm)**	**GF (μm)**	**FBS (μm)**	**GF (μm)**
Saracatinib (μM)	0	525.7 ± 32.1	381.6 ± 89.2 (^b^*p* < 0.001)	585.3 ± 32.5	595.8 ± 59.5 (^b^*p* = 0.2723)
	0.1	522.7 ± 25.9	407.5 ± 84.8	574.0 ± 24.7	586.9 ± 55.8
	1	543.8 ± 52.6	377.7 ± 83.8	585.4 ± 31.4	579.6 ± 54.2
	10	372.5 ± 24.3 (^a^*p* < 0.001)	359.5 ± 53.1	405.4 ± 34.7 (^a^*p* < 0.001)	540.2 ± 53.4 (^a^*p* = 0.0059)
	100	118.9 ± 28.9	160.9 ± 29.0 (^a^*p* < 0.001)	141.3 ± 29.9	208.6 ± 30.9
**B (Fold Change)**			**Wild-Type SNU-C5**		**Sox2-Upregulated SNU-C5**
		**FBS**	**GF**	**FBS (μm)**	**GF**
Day 5	DMSO	1.00 ± 0.04	1.00 ± 0.04	1.00 ± 0.04	1.00 ± 0.04
Day 10		1.66 ± 0.07	0.94 ± 0.10	1.62 ± 0.07 (^a^*p* = 0.0557)	1.02 ± 0.18 (^a^*p* = 0.0563)
Day 15		2.35 ± 0.16	1.17 ± 0.15	2.11 ± 0.015 (^a^*p* < 0.001)	1.40 ± 0.11 (^a^*p* = 0.0011)
Day 5	Saracatinib (30 μM)	0.76 ± 0.03	0.84 ± 0.07	0.72 ± 0.02 (^a^*p* < 0.001)	0.88 ± 0.06 (^a^*p* = 0.0431)
Day 10		0.99 ± 0.07	0.88 ± 0.12	0.88 ± 0.07 (^a^*p* < 0.001)	0.96 ± 0.06 (^a^*p* = 0.0168)
Day 15		1.19 ± 0.11	0.90 ± 0.18	1.05 ± 0.12 (^a^*p* = 0.0010)	1.07 ± 0.014 (^a^*p* = 0.0190)
		**Wild-Type SNU-C5/5-FUR**		**Sox2-Upregulated SNU-C5/5-FUR**	
		**FBS**	**GF**	**FBS**	**GF**
Day 5	DMSO	1.00 ± 0.04	1.00 ± 0.04	1.00 ± 0.07	1.00 ± 0.03
Day 10		1.53 ± 0.07	1.19 ± 0.06	1.53 ± 0.11 (^c^*p* = 0.4559)	1.38 ± 0.06 (^c^*p* < 0.001)
Day 15		2.09 ± 0.13	1.44 ± 0.24	2.01 ± 0.17 (^c^*p* = 0.1010)	1.79 ± 0.16 (^c^*p* < 0.001)
Day 5	Saracatinib (30 μM)	0.78 ± 0.02	0.85 ± 0.02	0.72 ± 0.04 (^c^*p* < 0.001)	0.83 ± 0.04 (^c^*p* = 0.0434)
Day 10		0.84 ± 0.04	0.96 ± 0.05	0.81 ± 0.05 (^c^*p* = 0.0157)	0.97 ± 0.07 (^c^*p* = 0.3339)
Day 15		0.93 ± 0.06	1.01 ± 0.06	0.87 ± 0.05 (^c^*p* = 0.0021)	1.09 ± 0.08 (^c^*p* = 0.0019)

Note: Results are expressed as mean ± SD. ^a^p, vs. DMSO; ^b^p, vs. environments; ^c^p, WT vs. Sox2-upregulated in same environment.

Saracatinib inhibited spheroid growth in both wild-type and Sox2-upregulated cells under both FBS- and GF-supplemented conditions (Table 4, Fig. 5B,C). Up to day 15, spheroid expansion patterns were comparable between wild-type and Sox2-upregulated cells. However, under GF-supplemented conditions, Sox2-upregulated spheroids were significantly larger than those derived from wild-type spheroid in both SNU-C5 (*p* < 0.001) and SNU-C5/5-FUR cells (*p* < 0.001).

## 4 Discussion

This study demonstrates that saracatinib induces apoptosis and inhibits migration in CRC cells, regardless of acquired 5-FU resistance. This finding is consistent with previous report indicating that saracatinib does not alter the efficacy of 5-FU [20]. In CRCs, Src expression is regulated by MAPKs pathways [15,16] and, to a lesser extent, by EGFR and Akt signaling [17]. In the present study, saracatinib exerted its’ *in vitro* anti-cancer effects primarily through inhibition of p38 and ERK signaling. These inhibitory effects were less prominent in 3D cultures. Saracatinib also markedly reduced EGFR expression in both cell types under 2D and 3D conditions, which was accompanied by decreased Akt activity in 2D cultures as reported the Src-EGFR-Akt cascade [21]. Saracatinib suppressed spheroid formation regardless of 5-FU resistance and resulted in inefficient spheroid formation under GF-supplemented conditions in both cell types, consistent with previous findings [12]. Except for Sox2, CSC-associated markers were inhibited by saracatinib in 2D cultures. In spheroids, expression level of pan-Ras and Klf4 were increased in both cell lines, in agreement with our previous results [12].

The unexpected upregulation of Sox2 following saracatinib treatment was further examined using spheroid formation assay. Under GF-supplemented conditions, spheroids derived from Sox2-upregulated CRC cells exhibited enhanced growth compared with those derived from wild-type cells, particularly in the SNU-C5/5-FUR cells. Src has been reported to upregulate Sox2, cMyc, and Nanog [22], while inhibition of the Src-EGFR-Akt pathway can reduce Sox2 expression [23]. In addition, Src activation can stimulate Akt signaling and subsequently promote Sox2-mediated CSC phenotypes in lung cancer [24]. Collectively, these findings support Sox2 as a downstream target of the Src-EGFR-Akt signaling axis.

Accumulating evidence also suggests crosstalk between Sox2 and p38 signaling in multiple cancer types. Reduced p38 activation accompanied by elevated Sox2 expression was observed in the present study, supporting the hypothesis that p38 inactivation may contribute to CSC-associated traits. Furthermore, p38-mediated phosphorylation of Sox2 at Ser251 has been shown to confer resistance to BRAF inhibitors by enhancing Sox2 binding to the promotor regions and increasing ABCG2 transcription in melanoma [25]. In our study, saracatinib markedly reduced CD44 expression in CRC cells in 2D and 3D cultures. Src kinase inhibition has also been reported to reduce Sox2 and the CD44^high^ cell populations in breast cancer [26]. Although CD44+ spheroids exhibit Sox2 with reduced p38 activity [18], EMT-induced spheroids in CRC cells did not demonstrate increased Sox2 expression in our experiment. Taken together, these findings suggest that inactivation of p38-regulated Sox2 may provide a mechanistic link to drug resistance via ABCG2 upregulation, particularly in SNU-C5/5-FUR cells.

We also considered whether spheroid formation depends on microenvironmental supplementation with FBS or GF. Compared with GF-supplemented conditions, FBS-supplemented spheroid formation represents an effective culture method for CSC enrichment in CRC cells [12]. This effect may be partially attributed to FBS-derived soluble E-cadherin, which can act as an EGF-like oncogene signal. In the present study, spheroid formation alone did not upregulate Sox2, consistent with our previous report [12]. Taken together, in GF-rich microenvironments, the enhanced drug efflux capacity of SNU-C5/5-FUR cells may, at least in part, facilitate saracatinib-induced Sox2 upregulation and promote a resistant phenotype. Because unintended promotion of CSC-associated functions may limit therapeutic efficacy, combination strategies have been proposed, including the use of Src kinase and MAPKs inhibitors [27,28]. Our findings suggest that combining saracatinib with p38 inhibitors and/or Sox2-targeting approaches may represent a promising strategy to overcome drug resistance in CRCs.

This study has several limitations. First, spheroid formation under GF-supplemented conditions yielded limited evidence, spheroids smaller than 100 μm may be insufficient for robust functional validation. Second, EMT-dependent regulation of Sox2 was not directly assessed. Future studies should therefore include systematic evaluation of EMT markers using rigorous experimental approaches. Finally, additional studies are required to examine the effects of Sox2-specific inhibition, including combination treatment with saracatinib, in CRC cells. Although the development of Sox2-specific inhibitors has been limited—largely because Sox2 is a transcription factor and therefore a challenging target for drug design [29]—a promising candidate targeting Sox2-driven CSCs has recently been reported [30]. These advances warrant further investigation using cell models capable of robust GF-dependent spheroid formation.

## 5 Conclusion

**Figure 6:**
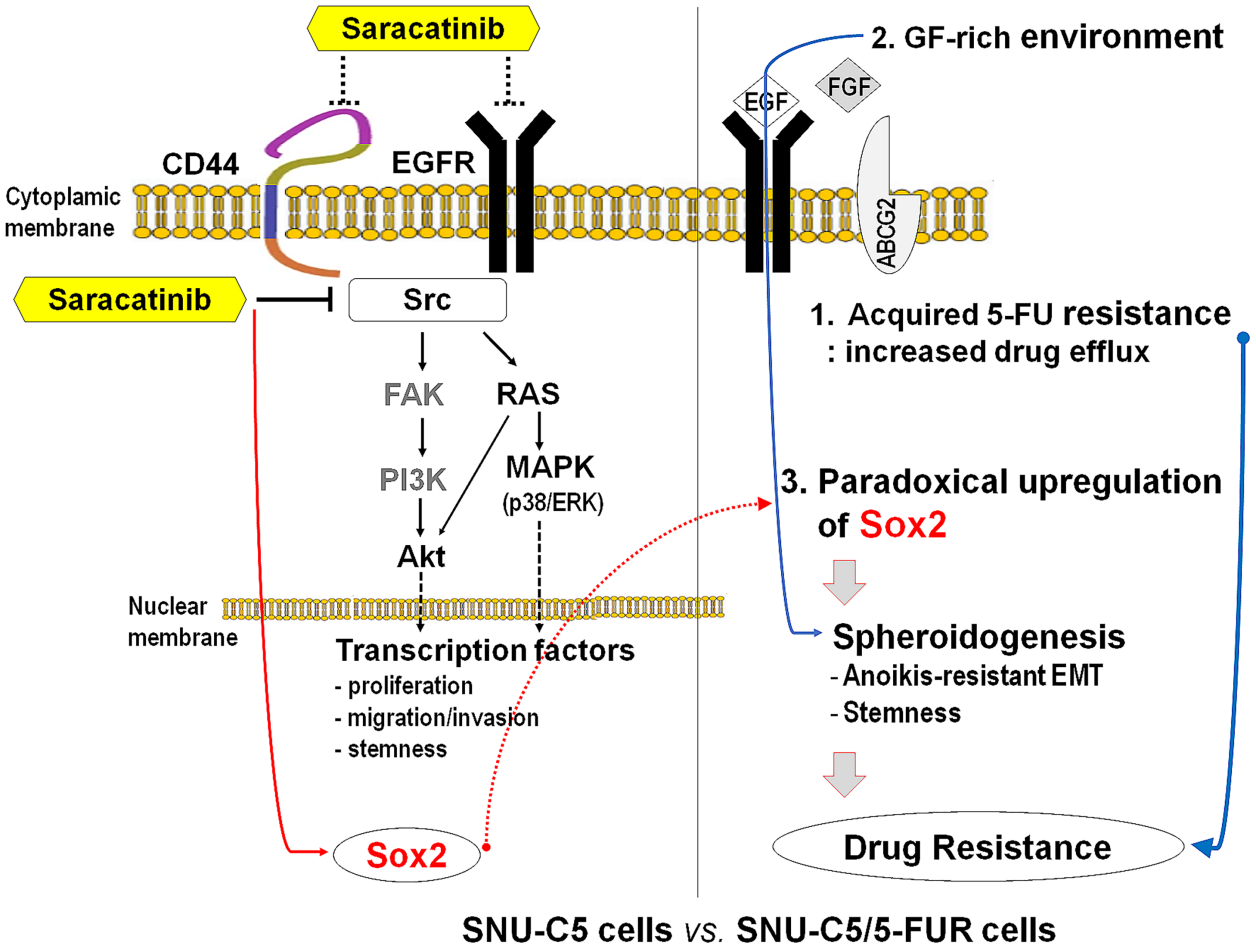
Schematic representation of the anti-cancer effects of saracatinib in colorectal cancer cells. 5-FU, 5-fluorouracil; ABCG2, ATP-binding cassette subfamily G member 2; Akt, protein kinase B; CD, cluster of differentiation; EGF, epidermal growth factor; EGFR, EGF receptor; EMT, epithelial-mesenchymal transition; ERK, extracellular signal-regulated protein kinase; FAK, focal adhesion kinase; FGF, fibroblast growth factor; GF, growth factor; MAPK, mitogen activated protein kinase; PI3K, phosphoinositide 3-kinase or phosphatidylinositol-3 kinase; RAS, rat sarcoma; SNU-C5/5-FUR, 5-FU-resistance acquired SNU-C5.

This study demonstrates that anti-cancer effects of saracatinib in SNU-C5 and SNU-C5/5-FUR cells are independent of 5-FU-resistance and are primarily mediated through modulation of CSC-associated markers and inhibition of MAPKs and EGFR signaling pathways. Saracatinib significantly suppressed spheroid formation. However, Sox2-upregulated CRC cells formed larger spheroids than wild-type cells, particularly under GF-supplemented conditions in ABCG2-upregulated SNU-C5/5-FUR cells (Fig. 6). These findings suggest that paradoxical Sox2 upregulation induced by saracatinib in specific microenvironments may contribute to drug resistance or recurrence in CRCs. Further studies are required to clarify the underlying mechanisms of saracatinib, particularly in the context of the tumor microenvironment.

## Data Availability

The datasets generated during and/or analyzed during the current study are available from the corresponding author upon reasonable request.

## Abbreviations

CRC: Colorectal cancer
5-FU: 5-fluorouracil
Akt: Protein kinase B
ERK: Extracellular signal-regulated protein kinase
SNU-C5/5-FUR: 5-fluorouracil-resistance acquired SNU-C5
ABCG2: ATP-binding cassette subfamily G member 2
CSC: Cancer stem cell
CD: Cluster of differentiation
Oct4: Octamer binding transcription factor-4
Sox2: Sex determining region Y-box-2
Klf4: Krüppel-like factor 4
EMT: Epithelial-mesenchymal transition
3D: 3-dimensional
GF: Growth factor
FBS: Fetal bovine serum
MAPK: Mitogen-activated protein kinase
EGF: Epidermal growth factor
EGFR: EGF receptor
bFGF: Basic fibroblast growth factor
ANOVA: One-way analysis of variance
